# Transient complete heart block during early ambulation associated with thoracic epidural bupivacaine infusion in the intensive care unit: a case report

**DOI:** 10.3389/fmed.2026.1829484

**Published:** 2026-05-14

**Authors:** Harshkumar B. Patel, Jason Ho, Andrew M. Blakely, Daniel S. Chertow

**Affiliations:** 1 Department of Critical Care Medicine, Clinical Center, National Institutes of Health, Bethesda, MD, United States; 2 Critical Care Medicine Branch, National Heart Lung and Blood Institute, National Institutes of Health, Bethesda, MD, United States; 3 Surgical Oncology Program, Center for Cancer Research, National Cancer Institute, National Institutes of Health, Bethesda, MD, United States; 4 Laboratory of Virology, National Institute of Allergy and Infectious Diseases, National Institutes of Health, Hamilton, MT, United States

**Keywords:** bupivacaine, case report, epidural analgesia, heart block, postoperative complication

## Abstract

**Background:**

Thoracic epidural analgesia is widely used for postoperative pain control and is generally considered safe. Rare but serious cardiovascular complications related to neuraxial sympathetic blockade have been reported.

**Case summary:**

We describe a postoperative intensive care unit patient who developed transient complete heart block during early ambulation while receiving a continuous thoracic epidural bupivacaine infusion. Telemetry demonstrated abrupt progression from sinus rhythm to high-grade atrioventricular block with ventricular standstill lasting approximately seven seconds, coinciding with hypotension and vagal symptoms. The episode resolved promptly with supportive care and interruption of the epidural infusion. No recurrent arrhythmias occurred after resumption of analgesia at a lower bupivacaine concentration.

**Conclusion:**

This case highlights a rare but clinically significant complication of thoracic epidural bupivacaine infusion and underscores how local anesthetic effects, in combination with postoperative autonomic imbalance, may precipitate transient high-grade atrioventricular block.

## Introduction

Continuous epidural analgesia is frequently used for postoperative pain control and is generally well tolerated, with well-established benefits. Large clinical trials and observational studies have demonstrated the overall safety and postoperative advantages of epidural techniques when appropriately managed, contributing to their widespread use in both surgical and critical care settings (1, 2).

Despite this favorable safety profile, neuraxial blockade can produce significant cardiovascular effects through interruption of sympathetic outflow, particularly in the early postoperative period when patients may be physiologically vulnerable. Blockade of thoracic sympathetic fibers may result in vasodilation, reduced venous return, and attenuation of cardiac chronotropic and dromotropic responses, thereby predisposing patients to hypotension and bradycardia (3). While hemodynamic effects are common, severe bradyarrhythmias, high-grade atrioventricular (AV) block, and even transient asystole have been infrequently reported.

We describe a case of transient complete heart block occurring during early mobilization in an intensive care unit (ICU) patient receiving a continuous thoracic epidural bupivacaine infusion, highlighting the mechanistic role of neuraxial sympathetic blockade and vagally-mediated reflexes.

## Case presentation

A 38-year-old man with years of vague abdominal pain was found to have peritoneal mesothelioma with a favorable peritoneal cancer index (PCI) on diagnostic laparoscopy and was referred to our center for surgical management. He underwent extensive cytoreductive surgery (CRS), achieving a completeness of cytoreduction (CC) score of 0, including complete pelvic and right diaphragm peritoneal stripping, low anterior resection, and radical right orchiectomy with cisplatin-based hyperthermic intraperitoneal chemotherapy (HIPEC) (4). He was subsequently admitted to the ICU for routine postoperative care. The patient had no prior history of cardiac disease, conduction abnormalities, or syncope and was not receiving AV nodal blocking medications. Baseline telemetry and preoperative 12-lead electrocardiogram demonstrated normal sinus rhythm.

Postoperative analgesia was provided via a thoracic epidural catheter placed at the T8–T9 interspace. The patient (weight ~75 kg) received patient-controlled epidural analgesia (PCEA) consisting of bupivacaine 0.125% (1.25 mg/mL) with fentanyl 2 mcg/mL. The infusion was administered at a basal rate of 12 to 16 mL/h (15 to 20 mg/h of bupivacaine), with patient-controlled 4 mL boluses (5 mg bupivacaine) available every 20 min as needed. In addition, to control pain not relieved by the PCEA, patient was receiving acetaminophen 1 gm every eight hours via intravenous route. Intravenous fentanyl was also available for breakthrough pain.

On postoperative day one, during early mobilization with physical therapy and transition to a seated position, the patient developed acute nausea, dizziness, and hypotension coinciding with a bowel movement. Continuous telemetry captured sinus rhythm with abrupt development of high-grade AV block, characterized by multiple non-conducted P waves, followed by complete AV block with ventricular standstill lasting approximately 7 s (Figure 1). Atrial activity persisted throughout the event. The patient remained conscious but was markedly symptomatic with transient drop in blood pressure as shown in Figure 1. Immediately preceding the event, patient had heart rate of 71 beats per minute and mean arterial blood pressure of 69 mmHg, as recorded by the telemetry.

**Figure 1:**
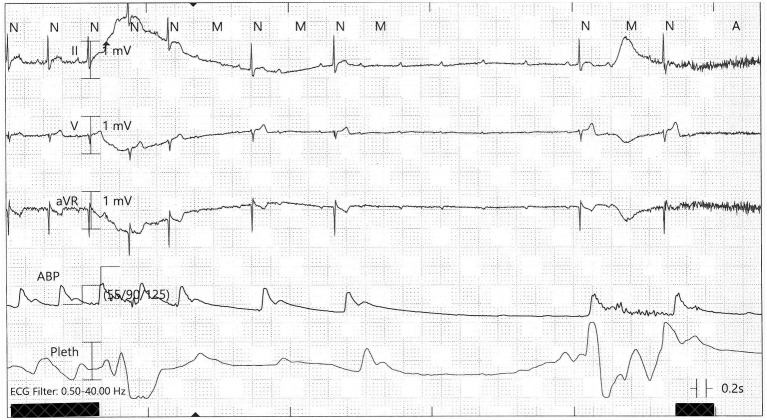
Continuous ICU telemetry demonstrating transient complete atrioventricular block simultaneous surface ECG leads (II, V, aVR) and invasive arterial blood pressure (ABP) tracing demonstrate abrupt onset of complete atrioventricular dissociation, accompanied by a marked reduction in arterial blood pressure.

The patient was immediately returned to the supine position, and the epidural infusion was paused in the setting of hypotension. Despite prompt return to normal rhythm with heart rate of around 70 beats per minute and restoration of blood pressure to mean arterial blood pressure of greater than 65 mmHg, intravenous bolus of lactated Ringer’s solution of 1 liter was administered. Telemetry after the event showed normal sinus rhythm without residual conduction abnormalities. A 12-lead electrocardiogram showed sinus rhythm without ischemic changes. Laboratory evaluation, including serum electrolytes, was unremarkable.

Following multidisciplinary discussion, the epidural infusion was resumed at a halved bupivacaine concentration without recurrence of bradyarrhythmia. The remainder of the ICU course was uncomplicated. Figure 2 highlights timeline of clinical course.

**Figure 2:**
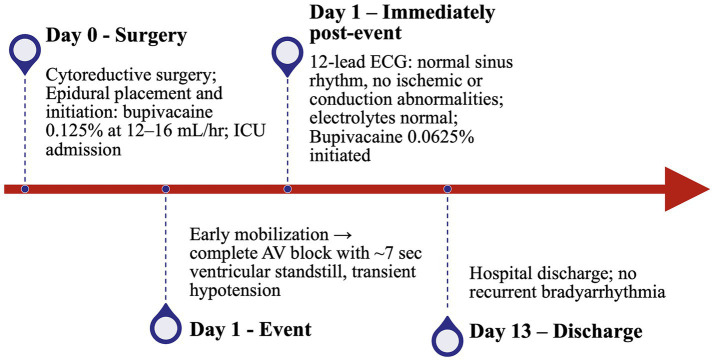
Timeline of clinical course.

## Discussion

Thoracic epidural analgesia is widely regarded as safe and is incorporated as a strong recommendation in the CRS/HIPEC enhanced recovery after surgery (ERAS) pathways due to its postoperative benefits, including effective pain control with reduced opioid utilization, earlier return of bowel function, and potential to reduce postoperative pulmonary complications (1, 2). However, this case highlights a rare but potentially serious cardiovascular complication attributable to neuraxial sympathetic blockade and autonomic imbalance.

The cardiovascular effects of neuraxial anesthesia are largely mediated through blockade of sympathetic efferent fibers (5). Cardiac sympathetic innervation arises primarily from the T1–T4 spinal segments; however, accumulating evidence suggests that the anatomy of the human cardiac autonomic nervous system is variable and may extend both above and below above these segments (6). Thoracic epidural blockade can attenuate chronotropic and dromotropic responses by reducing sympathetic tone to the sinoatrial and atrioventricular nodes and subsequently impair compensatory responses to physiologic stress (7). Reduced coronary perfusion to sinoatrial and atrioventricular nodes can also induce transient conduction abnormalities. The epidural catheter was placed at the T8–T9 level, and the infusion was maintained at the upper end of the prescribed dosage range, with additional patient-controlled boluses contributing to overall local anesthetic exposure. Although this level is below the classic T1–T4 cardiac accelerator fibers, cephalad spread of local anesthetic may still result in partial blockade of cardiac sympathetic outflow, particularly in the setting of higher infusion volumes (6).

The episode occurred during early mobilization with transition to a seated position and coincided with a bowel movement, both potent vagal stimuli. The Bezold-Jarisch reflex and other vagally-mediated cardiovascular reflexes are well-described in anesthetic practice and are characterized by paradoxical bradycardia and hypotension in response to decreased ventricular filling or heightened vagal tone (8). Additionally, reduced venous return and preload from vasodilation combined with relative hypovolemia due to insensible losses following a prolonged operation can further diminish cardiac output and potentiate reflex responses (5). Moreover, the abdominal viscerae are richly innervated by the vagal afferent fibers, and surgical manipulation or postoperative gastrointestinal activity can also directly affect vagal tone (9). In the setting of neuraxial sympathetic blockade, unopposed parasympathetic activity may precipitate profound bradycardia, sinus arrest, or high-grade AV block.

While the temporal association with mobilization or a bowel movement may suggest a purely vagal trigger, the severity of the conduction failure suggests an underlying pharmacologic substrate. Bupivacaine is uniquely cardiotoxic, as it blocks inactivated Na^+^ channels in a time- and voltage-dependent fashion with high affinity. However, it dissociates slowly during diastole, and this ‘fast-in-slow-out’ effect on myocardium can lead to slowed conduction, ventricular arrhythmias, and myocardial depression at toxic plasma concentrations (10). In this case, the infusion consisted of low-concentration bupivacaine delivered via continuous epidural administration for analgesia rather than bolus dosing often used for anesthesia (11). Plasma concentrations with continuous epidural infusion can approach the threshold for toxicity due to bupivacaine’s long half-life and rapid systemic absorption (12). Given this, local anesthetic systemic toxicity (LAST) was considered in the differential diagnosis. However, the observed rhythm with abrupt development of high-grade AV block without QRS widening, absence of neurologic symptoms such as tinnitus, metallic taste, perioral numbness, or seizures, and rapid resolution, suggest that LAST is unlikely to be the primary mechanism in this case (13, 14). Furthermore, the synergy between bupivacaine-induced Na^+^ channel blockade and neuraxial mediated cardiac sympathetic blunting can create a “perfect storm” for high-grade AV block. Additionally, the potential contributory effects of epidural and systemic opioids warrant consideration. Fentanyl, commonly co-administered with local anesthetics, has been associated with modulation of autonomic tone and may enhance vagally-mediated responses, although its role in precipitating significant conduction disturbances remains unclear (15). No intravenous opioid boluses were administered preceding the event in this case.

The transient nature of the event, absence of baseline conduction disease, and complete recovery without recurrence further support a functional rather than structural conduction abnormality, although undiagnosed sick sinus syndrome or nodal disease cannot be completely excluded. While absence of measured plasma bupivacaine levels and the inherent inability of a single case to establish causality remain limitations of this case, several case reports describe transient complete AV block or asystole occurring during continuous epidural local anesthetic infusion either for analgesia or for anesthesia (16–19). These reinforce the role of sympathetic blockade and autonomic imbalance, rather than local anesthetic systemic toxicity, in predisposing susceptible patients to severe conduction disturbances.

This case also underscores the importance of continuous telemetry monitoring in patients receiving thoracic epidural analgesia, especially during early postoperative mobilization. The immediate postoperative period represents a particularly vulnerable time for autonomic instability. Surgical stress, volume shifts, residual anesthetic effects, pain, and early mobilization all contribute to dynamic changes in autonomic tone (20). Thoracic epidural analgesia, while beneficial for pain control and pulmonary function, may blunt compensatory sympathetic responses to these stressors, and hence careful selection of pharmacologic agents and dose control is paramount to prevent adverse effects.

From a broader perspective, this case contributes to the growing body of literature emphasizing that severe bradyarrhythmias associated with neuraxial analgesia are rare but real phenomena. Importantly, such events should not automatically be attributed to primary cardiac pathology or systemic toxicity without careful consideration of neuraxial physiology and contextual triggers. As enhanced recovery pathways increasingly emphasize early mobilization, clinicians should maintain heightened vigilance for autonomic complications in patients with thoracic epidurals, particularly in the immediate postoperative period.

## Conclusion

Thoracic epidural bupivacaine infusion can rarely be associated with transient complete heart block. Awareness of this potential complication, resulting from the complex interplay between the anesthetic drug, post operative physiology, and cardiovascular reflexes is essential to prevent adverse outcomes.

## Patient perspective

The episode was brief but unsettling, especially since it occurred during what felt like a routine part of recovery. Although it resolved quickly, it seemed to influence the remainder of my hospital stay. Afterward, my pain management plan was adjusted, and I found the following days more uncomfortable than I had expected. This made mobilization and rehabilitation more challenging and, at times, discouraging. I understood that decisions were being made cautiously in response to what had happened. From my perspective, the experience showed how an unexpected complication, even one that resolves, can affect many aspects of recovery and how important shared decision-making is.

## Data Availability

The original contributions presented in the study are included in the article/supplementary material, further inquiries can be directed to the corresponding author/s.
